# Identification of the First SARS-CoV-2 Lineage B.1.1.529 Virus Detected in Europe

**DOI:** 10.1128/mra.01161-21

**Published:** 2022-02-03

**Authors:** Bert Vanmechelen, Anne-Sophie Logist, Tony Wawina-Bokalanga, Jens Verlinden, Joan Martí-Carreras, Caspar Geenen, Bram Slechten, Lize Cuypers, Emmanuel André, Guy Baele, Piet Maes

**Affiliations:** a KU Leuven, Department of Microbiology, Immunology, and Transplantation, Laboratory of Clinical and Epidemiological Virology, Rega Institute for Medical Research, Leuven, Belgium; b KU Leuven, Department of Microbiology, Immunology, and Transplantation, Laboratory of Clinical Microbiology, National Reference Laboratories for COVID-19, Leuven, Belgium; c UZ Leuven, Department of Laboratory Medicine, Leuven, Belgium; DOE Joint Genome Institute

## Abstract

We report the complete genome sequence of a severe acute respiratory syndrome coronavirus 2 (SARS-CoV-2) Omicron variant (lineage B.1.1.529) from a Belgian patient with a history of recent travel to Egypt. At the time of writing, this genome constituted the first confirmed case of an infection with the Omicron variant in Europe.

## ANNOUNCEMENT

In December 2019, a novel betacoronavirus, severe acute respiratory syndrome coronavirus 2 (SARS-CoV-2) (genus *Betacoronavirus*, subfamily *Orthocoronavirinae*, family *Coronaviridae*), was identified in Wuhan, China (1, 2). Following its spread to the rest of the world, many unique mutations in the virus genome have been reported. The biological advantages conferred by some of these mutations have resulted in the establishment of specific genetic lineages that have largely outcompeted the original strain (3). Lineages that are characterized by increased transmissibility or virulence or that decrease the effectiveness of available countermeasures are classified by the WHO as variants of concern (VOCs) (4). On 26 November 2021, 2 days after it was reported for the first time in South Africa, a new lineage (B.1.1.529) was classified as variant Omicron, the fifth recognized VOC (5). Here, we report the genome sequence of the first reported Omicron variant in Europe.

The sample was collected as part of routine COVID-19 diagnostics and selected for whole-genome sequencing following an S gene dropout in quantitative PCR (qPCR) testing (TaqPath COVID-19 CE-IVD reverse transcription [RT]-PCR kit; Thermo Fisher Scientific). This work was reviewed and approved by the KU/UZ Leuven Clinical Trial and Ethical review board (approval number S66037), which allows us to use patient samples for research and lookup and use specific metadata from the patient. The patient returned from Egypt to Belgium on 11 November 2021 and tested negative 3 days before travelling, as well as repeatedly testing negative during quarantine after arrival in Belgium. The patient developed mild symptoms 10 days after arrival and tested positive for SARS-CoV-2 on 24 November 2021. RNA was extracted from a nasopharyngeal swab sample using the QIAamp viral RNA minikit (Qiagen, Hilden, Germany), after which a barcoded Nanopore sequencing library was prepared using the COVID Midnight midikit (C19MIDI; Oxford Nanopore Technologies), which uses a tiled amplicon approach (∼1,200-bp amplicons) to amplify the SARS-CoV-2 genome. The library was sequenced for 72 h on an R9.4.1 flow cell. Base calling and demultiplexing were done using the GridION built-in MinKNOW software (v21.05.25), and the resulting reads were processed using the ARTIC bioinformatics pipeline v1.1.3 (6).

The genome sequence obtained has a total length of 29,684 nucleotides and a GC content of 38%, with an average coverage depth of 1,501×. Because the genome termini are not covered by the amplicons used, the 54 first (5′) and 151 last (3′) nucleotides, compared to the SARS-CoV-2 reference genome (GenBank accession number NC_045512.2), are not present in this sequence. Nextclade v1.10.0 was used for comparison with the RefSeq sequence (7). This genome contains 54 mutations and 39 indels (Table 1), compared to the RefSeq sequence, highlighting the strong divergence of this novel variant.

**TABLE 1:** Mutations of SARS-CoV-2 strain hCoV-19/Belgium/rega-20174/2021 in comparison with the reference strain (GenBank accession number NC_045512.2)

Gene or region^*a*^	Nucleotide position(s)	Amino acid change^*b*^	Coding sequence codon position(s)^*b*^	Nucleotide change^*b*^
ORF1a	241	—	—	C to T
	2832	K to R	856	A to G
	3037	—	—	C to T
	5386	—	—	T to G
	6513–6515	S deletion	2083	—
	8393	A to T	2710	G to A
	10029	T to I	3255	C to T
	10449	P to H	3395	C to A
	11285–11293	LSG deletion	3674–3676	—
	11537	I to V	3758	A to G
	13195	—	—	T to C
ORF1b	14408	P to L	314	C to T
	15240	—	—	C to T
	18163	I to V	1566	A to G
S	21762	—	—	C to T
	21765–21770	HV deletion	69–70	—
	21846	T to I	95	C to T
	21987–21995	GVY deletion	142–144	—
	22194–22196	N deletion	211	—
	22578	G to D	339	G to A
	22673–22674	S to L	371	TC to CT
	22679	S to P	373	T to C
	22686	S to F	375	C to T
	22813	K to N	417	G to T
	22882	N to K	440	T to G
	22898	G to S	446	G to A
	22992	S to N	477	G to A
	22995	T to K	478	C to A
	23013	E to A	484	A to C
	23040	Q to R	493	A to G
	23048	G to S	496	G to A
	23055	Q to R	498	A to G
	23063	N to Y	501	A to T
	23075	Y to H	505	T to C
	23202	T to K	547	C to A
	23403	D to G	614	A to G
	23525	H to Y	655	C to T
	23599	N to K	679	T to G
	23604	P to H	681	C to A
	23854	N to K	764	C to A
	23948	D to Y	796	G to T
	24130	N to K	856	C to A
	24424	Q to H	954	A to T
	24469	N to K	969	T to A
	24503	L to F	981	C to T
	25000	—	—	C to T
ORF3a	25584	—	—	C to T
E	26270	T to I	9	C to T
M	26530	D to G	3	A to G
	26577	Q to E	19	C to G
	26709	A to T	63	G to A
ORF6	27259	—	—	A to C
ORF7b	27807	—	—	C to T
N	28271	—	—	A to T
	28311	P to L	13	C to T
	28362–28370	ERS deletion	31–33	—
ORF9b	28362–28370	ENA deletion	27–29	—
N	28881–28883	RG to KR	203–204	GGG to AAC

a ORF, open reading frame.

b —, no amino acid or codon change occurred, and no nucleotide change occurred when a deletion was detected.

**FIG 1:**
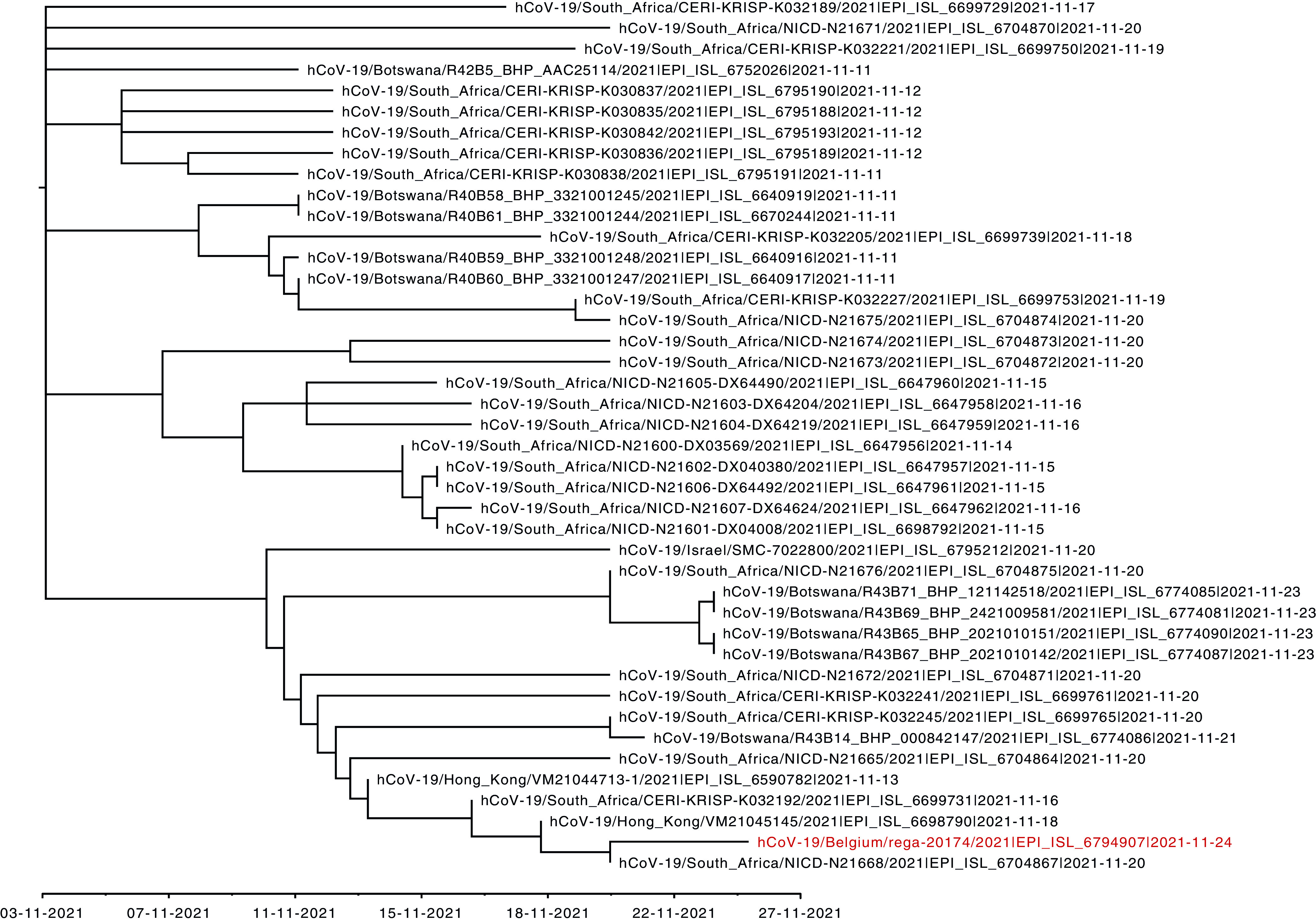
Time-calibrated phylogenetic tree of strain hCoV-19/Belgium/rega-20174/2021 (highlighted in red) in a clade of the most closely related Omicron variant sequences (black). EPI_ISL_ numbers in the entries are GISAID accession numbers.

A phylogenetic analysis was performed using all available B.1.1.529 genomes in GISAID on 27 November 2021, along with a few representative genomes of the different VOCs (to root the phylogeny). MAFFT v7.475 was used to align the genome sequences, and the initial tree was generated using IQ-TREE v2.1.3 with automated model selection (8, 9). Following inspection of the resulting phylogeny using TempEst v1.5.3, nine outliers were removed to allow the subsequent estimation of a time-calibrated phylogeny using TreeTime v0.8.1 (10, 11). The resulting tree was visualized using FigTree v1.4.4 (Fig. 1). The phylogenetic analysis shows that the sequence reported here, hCoV-19/Belgium/rega-20174/2021, clusters as part of a larger clade with South African cases, the two previously confirmed cases from Hong Kong, and the one confirmed case from Israel.

### Data availability.

This sequence has been deposited in GISAID (accession number EPI_ISL_6794907) and GenBank (accession number OL672836). The accession numbers for the raw sequencing reads in the NCBI Sequence Read Archive (SRA) are PRJNA784547 and SRR17066006.
